# Vaginal progesterone on the prevention of preterm birth and neonatal complications in high risk women: A randomized placebo-controlled double-blind study

**Published:** 2016-05

**Authors:** Azam Azargoon, Raheb Ghorbani, Fereshteh Aslebahar

**Affiliations:** 1 *Abnormal Uterine Bleeding Research Center, Semnan University of Medical Sciences, Semnan, Iran.*; 2 *Department of Infertility, Amir-AL-Momenin Hospital, Semnan University of Medical Sciences, Semnan, Iran. *; 3 *Social Determinants of Health Research Center, Department of Community Medicine, Faculty of Medicine, Semnan University of Medical Sciences, Semnan, Iran.*

**Keywords:** *Premature birth*, *Prevention*, *Progesterone*, *Randomized controlled trial*

## Abstract

**Background:**

Preterm birth is the major cause of neonatal mortality and morbidity.

**Objective:**

The aim of this study was to evaluate the effect of prophylactic vaginal progesterone on decreasing preterm birth rate and neonatal complications in a high-risk population.

**Materials and Methods:**

A randomized, double-blind, placebo-controlled study was performed on 100 high-risk singleton pregnancies. Vaginal suppository progesterone (400 mg) or placebo was administered daily between 16-22 wks to 36 wks of gestation. Progesterone (n=50) and placebo (n=50) groups were compared for incidence of preterm delivery and neonatal complications.

**Results:**

The preterm birth rate was 52%. Preterm birth rate before the 37 wks of gestation (68% vs. 36%: RR=1.89, 95% CI: 1.25-2.86) and also before the 34 wks of gestation (42% vs. 18%: RR=2.33, 95% CI: 1.19-4.58) in placebo group was significantly higher than progesterone group. Our study also showed that the administration of vaginal progesterone was associated with a significant reduction in the risk of birth weight ≤2500 gr, the rates of respiratory distress syndrome (RDS) and admission to the Neonatal Intensive Care Unit (NICU) in the progesterone group when compared with the placebo group. However, there was no significant difference between the two groups in terms of neonatal death, days of admission in NICU, intraventricular hemorrhage and necrotizing enterocolitis.

**Conclusion:**

Prophylactic vaginal progesterone reduced the rate of preterm delivery, the risk of a birth weight ≤2500 gr, the rates of RDS and admission to NICU in women who were at risk of preterm delivery.

## Introduction

Preterm birth (PTB), is defined as birth prior to 37 wks completed gestation (1). PTB incidence has been increased over recent years. It affects approximately 12% of births in the United States and it is implicated in approximately 2/3 of neonatal mortality (1-4). Despite improvements in neonatal care, PTB is the main cause of handicaps and long-term disabilities in children born without congenital anomalies (5). Therefore, PTBs prevention is the main goal of obstetric care. Despite using tocolytics, antibiotics and reduced activity there is not yet a definite method for prevention of PTB (6). The factors known for causing PTB include having a history of previous PTB, a short cervix, tobacco use, black race, low socioeconomic class, multiple births and extreme maternal age (7).

The first randomized controlled trial to study the effect of progesterone for the prevention of PTB among women at increased risk was published by Papiernik in 1970 (8). Afterwards, several studies were carried out to determine how effective progesterone was in preventing PTB, some of which reported positive results of using 17OH- progesterone caproate or vaginal progesterone in reducing the preterm delivery likelihood (9-12). On the contrary, some other of these studies observed no evidence of progesterone effectiveness in preterm delivery prevention (13, 14). 

The American College of Obstetricians and Gynecologists (ACOG) in 2009 and more recently Dodd and his colleagues in 2013 in Cochrane’s review concluded that although administration of progesterone for women with a previous history of PTB or with short cervix may reduce their risk of PTB, there are still unknown questions about the progesterone’s mechanism action, the optimal dose and route and progesterone impact on perinatal outcomes. Thus, they suggested performing further randomized controlled trials in this field (15, 16). 

In Iran, vaginal progesterone (400 mg) is normally used to support the luteal phase in ovulation induction and IVF cycles. Since this dose of progesterone has never been studied to prevent preterm delivery in women who run a high risk of it, we decided to take up this study. 

## Materials and methods

This randomized, double-blind, placebo-controlled study was carried out from November 2010 to April 2012 on 16-22 wks of singleton pregnancy women which referred to prenatal clinic of Amir-AL-Momenin Hospital. This study was supervised and approved by the Research Council and Ethical Committee of Semnan University of Medical Sciences. The patients were given sufficient information about the study, the way to use the medicine and the probable side-effects of the medicine and their informed consent was obtained. 

Women at high risk of preterm delivery were considered to be those who met at least one of the following criteria.

1- Women with a previous history of PTB (at least one PTB <37 wks).

2- Women with a previous history of PTB and cervical length ≤28 mm and who had already undergone cerclage surgery.

3- Women with uterine anomalies (including septate, unicornuate, bicornuate or didelphys uterus).

4- Pregnancies with uterine intramural myoma ≥7 cm.

In women with septate uterus, their septum was removed, using hystroscopy, before pregnancy. All these women underwent cerclage surgery during their 14-16 wks of gestation. All were Iranian mothers and none of them used drugs or smoked. 

The exclusion criteria were: clinical evidence indicating amniotic fluid infection, sensitivity to progesterone, any detection of anomalies in the fetus which could lead to its death, multiple births, polyhydramnious, intra uterine growth retardation, hyperthyroidism, gestational diabetes, blood pressure ≥140/90 mm Hg, heart disease, epilepsy and the use of anti-convulsive agents.

Gestational age was determined by an ultrasound scan done in the first trimester (before the 12 wks of gestation). The length of the cervix was measured by a sonograghist, using a vaginal ultrasound (Honda, Japan, probe 6.5 MHz) during the 14-18 wks of gestation. The patients whose length of cervix was ≤28 mm underwent a cerclage surgery, otherwise they just took progesterone or placebo.

106 women at risk of PTB were eligible to enter the study. Three of the participants were excluded from the study before the randomization (two cases of early abortions and one case of an intra-uterine fetal death in the 14 wks of gestation). The 103 remaining women were randomly divided into two groups of receiving progesterone treatment (group A) and placebo (group B) according to computerized list of randomized numbers. Vaginal suppository progesterone or placebo vaginal suppositories (400 mg) in identical packs were prepared by manufacturer (Aboryhan, pharmacy Tehran, Iran). 51 patients received progesterone and the remaining 52 patients received placebo vaginal suppository one capsule every night between 16-22 wks of gestation till 36 wks of gestation. Placebo was similar to suppository progesterone in shape and thickness. 

The patients (including, 1^st^ group: those with a previous history of PTB, 2^nd^ group: those with a history of PTB and a short cervix, 3^rd^ group: those with uterine anomalies and the 4^th^ group: those with uterine myomas) came to see the staff in charge at the clinic and in each group the patients received the drug administered to group A or B in order of their clinic appearance. Neither the staff nor the patient knew which drug was administered. The two groups were matched in terms of background factors such as age and risk factor of having previous PTB.

The uterine contractions of patients were checked by resident in prenatal clinic every 4 wks for the first 28 wks and then every 2 wks till 36 wks which were recorded in patient’s file. Preterm labor was defined as 5-6 regular contractions in 30 min associated with cervical changes, represented by a dilatation of more than 2 cm or the presence of progressive dilatation or effacement of cervix. In the case of such symptoms the patients were administered intravenous magnesium sulfate (the primary dose was 4 gr and then 2 gr/hr) for 12 hr.

All patients received two doses of 12 mg betamethasone intramuscularly within an interval of 24 hr, in the 28 wks of gestation and earlier if they developed preterm labor. The women who had received magnesium sulfate entered the study again when they were finished with it unless they had already given birth. PTB is defined as the baby being born alive before 37 wks when gestation is completed The primary outcome was PTB before the 37 wks of gestation and the secondary outcomes included PTB before the 34 wks of gestation, birth weight of less than 2500 gm and 1500 gm, neonatal death, rate of admission to the neonatal intensive care unit (NICU), the number of days of admission to NICU, respiratory distress syndrome(RDS), intracranial hemorrhage and necrotizing enterocolitis. 

**Statistical analysis**

Regarding the results of a pilot study, with α=0.05 and β=0.2, the required sample size for each group obtained was about 48, based on the compare mean or proportion formula in two independent populations.

All data were entered into the SPSS software (Version 16.0, © SPSS Inc.). Results were reported as mean±SD for quantitative variables and percentages for categorical variables. Statistical analysis were performed using Student's t-test (or Mann Whitney U test) and the  ^2 ^test (or Fisher's exact test). Also Relative Risk (RR) and its 95% Confidence Interval (CI) were calculated. p˂0.05 were considered statistically significant. We used Kolmogorov-Smirnov test for evaluation of normality.

## Results

**Figure 1:**
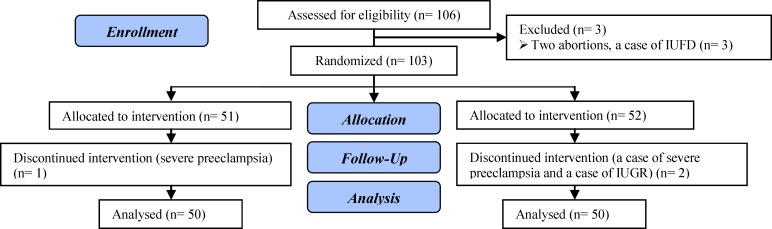
Flow diagram of the study

103 patients at risk of preterm delivery entered the study, 51 of whom were randomly put in progesterone group (group A) and 52 of them were placed in placebo group (group B). Later, 3 patients were withdrawn from study, 2 of them because of severe preeclamsia (one in each group) and one patient (from the placebo group) because of intra uterine growth retardation. Eventually the study was conducted on 100 patients (50 patients in each group) (Figure 1).

The people in both groups were compared in terms of age, the factors causing PTB and the length of cervix. No significant difference was observed between two groups (Table I). PTB rate was 52% in this study. PTB rate before the 37 wks of gestation (68% vs. 36%: RR=1.89, 95% CI: 1.25-2.86) and also before the 34 wks of gestation (42% vs. 18%: RR=2.33, 95% CI: 1.19-4.58) in placebo group was significantly higher than progesterone group.

The mean gestational age at birth was 36.5±3.8 wks for progesteroneand 33.6±4.5 wks for placebo group (p=0.001). The average birth weight was 2727±844 gm in progesterone and 2041±873 gm in placebo group (p=0.001). When the groups were studied separately, PTB rate before the 37wks (35.7% vs. 76%, RR=2.13, 95% CI: 1.24-3.66) and 34 wks of gestation (17.9% vs. 44%, RR=2.46, 95% CI: 1.02-6.12) was significantly lower for women with previous history of PTB in progesterone group than placebo group. However, with regard to women with short cervix and uterine anomalies, there were no significant differences between two groups in terms of PTB rate before the 37 and 34 wks of gestation (Table II).

Due to small number of women with uterine leiomyoma, we could not evaluate progesterone effect in this subgroup. About neonatal outcomes, there was a significant reduction in the risk of birth weight of less than 2500 gm or 1500 gm, the rates of RDS and admission to NICU in progesterone compared with placebo group. However, there was no significant difference between two groups in terms of neonatal death, days of admission to NICU, intraventricular hemorrhage and necrotizing enterocolitis (Table III). Administration of vaginal progesterone did not have any serious side-effects except for more vaginal discharges than usual, and in some cases vaginal irritation.

**Table I:** The baseline characteristics of patients

**Variables**		**Study group**		**p-value**
		**Progesterone**	**Placebo**	
Risk factors				
	Age*	25.4 ± 4.8	24.6 ± 4.9	0.291
	Previous preterm birth (PPTB)**	28 (56 %)	25 (50 %)	0.857
	PPTB and short cervix**	12 (24 %)	15 (30 %)	
	Uterine anomalies**	7 (14 %)	8 (16 %)	
	Uterine mommas**	3 (6 %)	2 (4 %)	
	History of one PPTB**	43 (86 %)	44 (88 %)	0.766
	History of two or more PPTB**	5 (10 %)	4 (8 %)	0.728
Cervical length (mm)*				
	All patients	31.2 ± 6.5	30.4 ± 6.3	0.545
	In women with PPTB	34.5 ± 2.4	34.2 ± 2.8	0.643
	In women with short cervix	21.2 ± 5	22.2 ± 4.5	0.606
	In women with uterine anomalies	32.7 ± 2.6	32.6 ± 2.4	0.946

Student’s t- test for age and cervical length; ^2^ test for the other variables (n=50).

* Data are presented as mean±SD.

** Data are presented as n (%).

**Table II:** Outcomes of pregnancy according to treatment assignment

**Outcomes**			**Study Group**			RR* ( 95 % CI )		**p-value**		
			**Progesterone**	**Placebo**						
All patients										
	Delivery ˂37 wks of gestation		18 (36 %)	34 (68 %)		1.89 (1.25–2.86)			0.001 a	
	Delivery ˂34 wks of gestation		9 (18 %)	21 (42 %)		2.33 (1.19–4.58)			0.009 a	
Women with PPTB										
	Delivery ˂37 wks of gestation	10 (35.7 %)		19 (76 %)	2.13 (1.24–3.66)			0.003 a		
	Delivery ˂34 wks of gestation	5 (17.9 %)		11 (44 %)	2.46 (1.02–6.12)			0.040 a		
Women with PPTB & short cervix										
	Delivery ˂37 wks of gestation		2 (16.7 %)	7 (46.7 %)			2.80(0.71–11.09)			0.107 a
	Delivery ˂ 34 wks of gestation		-	4 (26.7 %)			-			0.078 b
Women with uterine anomalies										
	Delivery ˂37 wks of gestation	6 (85.7 %)		7 (87.5 %)	1.02(0.68-1.52)			1.00 b		
	Delivery ˂34 wks of gestation	4 (57.1 %)		5 (62.5 %)	1.25(0.56-2.77)			1.00 b		

Data are presented as n (%).

RR: Relative Risk

CI: Confidence Interval

PPTB: Previous preterm birth

a χ^2 ^test

b with the use of Fisher exact test.

**Table III:** Fetal and neonatal outcomes according to maternal treatment groups

**Neonatal outcomes**	**Study Group**		**RR ( 95 % CI ) ** *	**p-value**
	**Progesterone**	**Placebo**		
Gestational age at birth (wks)*	36.5±3.8	33.6±4.5	-	0.001
Fetal weigh ˂2500 gm**	20 (40 %)	33 (66 %)	1.65(1.11–2.45)	0.009
Fetal weigh t˂1500gm**	5(10%)	18(36%)	3.6(1.45-8.94)	0.002
Mean neonatal weight (gm )*	2727 ± 844	2041±873	-	0.001
Neonatal mortality**	2 (4 %)	21 (42 %)	4.0 (0.89-17.91)	0.056
Fetal Respiratory distress syndrome**	10 (20 %)	21 (42 %)	2.1 (1.10–3.99)	0.017
Admission in NICU**	13 (26 %)	27 (54 %)	2.08(1.22–3.54)	0.004
Hospitalization in NICU (day)*	11.6±5.8	10.4±6.1	-	0.53
Intra ventricular hemorrhage**	5 (10 %)	10 (20 %)	2.0 (0.74–5.43)	0.161
Necrotizing enterocolitis**	4 (8 %)	9 (18 %)	2.25 (0.74–6.83)	0.137

RR: Relative Risk

CI: Confidence Interval

P-values were determined using Student’s t-test for comparison of continues variable, and χ^2^ for comparison of categorical variables, P-value< 0.05 was considered significant. (n=50)

* Data are presented as mean±SD.

** Data are presented as n (%).

## Discussion

The findings of this study showed that the administration of vaginal suppository progesterone (400 mg, daily) beginning at 16-22 wks of gestation and continued to 36 wks of gestation can significantly reduce the PTB rate before 37 and 34 wks of gestation among women at high risk of PTB. In addition, the rates of birth weight less than 2500 gm and 1500 gm, RDS and admission to NICU were correspondingly decreased among the infants of women assigned to this treatment. 

PTB rate was 52% in this study, whereas it varied from 21.1% to 52% in other studies (10, 12, 14, 17-20). The reason for this variation is the population under study in different studies. In our study, PTB rate was relatively high basically because 80% of participants had a previous history of PTB and they also carried other risk factors such as uterine anomalies or a short cervix. 

The mechanism of progesterone’s action in second and third trimester to prevent preterm delivery is not clear yet. Its effects on myometrium include: relaxation of smooth muscles, inhibiting the action of oxytocin and gap junction formation (21, 22). It also inhibits the synthesis of stimulatory prostaglandins and the expression of contraction-associated protein genes in myometrium (23). 

In some mammals, a systemic drop in progesterone and a rise in circulating estrogen heralds the onset of parturition (24). Although, this event does not occur in humans, there is evidence that local changes in progesterone level or progesterone to estrogen ratio in placenta, decidua and fetal membranes, might be responsible for initiation of labor (25). This might be true since the administration of progesterone antagonists such as mifepristone to women at term increases labor pain (26). Recently the important effects of progesterone on cervix have been pointed out, therefore it can have a role in prevention rather than PTB treatment. It is suggested that the decrease in local progesterone responsiveness, named functional withdrawal, precede cervical remodeling (softening, ripening and dilating). Probably one mechanism in this process is reduced expression of progesterone-responsive genes (27).

A number of studies have been carried out to determine the effect of vaginal or intramuscular progesterone on PTB prevention. In 2003, Meis and his colleagues conducted a clinical, randomized, double blind study on 463 patients with a previous history of PTB. They discovered that weekly injections of 250 mg 17-OH-progestrone caproate beginning at 16-20 wks of gestation and continued to 34 wks of gestation significantly reduced preterm delivery(PD) before 37, 35 and 34 wks of gestation. They also demonstrated that treatment with progesterone decreased rate of birth weight of ≤2500 gr, intraventricular hemorrhage, necrotizing enterocolitis and need for supplemental oxygen (10).

In another randomized clinical study, Da Fonseca *et al* showed that the administration of 100 mg vaginal progesterone to women with a PD history, cerclage, and uterine anomalies caused a reduction in preterm delivery rate before 34 wks of gestation and uterine contractions compared with placebo group (12). On the other hand, in 2007 O’Brien and his colleagues observed that the administration of vaginal progesterone gel (90 mg) compared to placebo did not reduce the rate of early PD (≤32 wks) or the frequency of neonatal morbidity and mortality in women with a spontaneous PB history (14). In another clinical trial in 2007 Borna *et al* reported on the use of vaginal progesterone (400 mg) for arrested preterm labor. In their study the mean latent time until delivery was significantly longer in progesterone group (12 days). They also observed that the risk of LBW and RDS were decreased among the infants of women assigned to this therapy compared to group which had not received any drug (28).

Ultimately in 2009 ACOG announced that progesterone may be effective in prevention of recurrent PD in women with a history of spontaneous PLB and recommended its use in such cases, however they expressed uncertainties as to its mechanism of action, its ideal form, rout of administration, its optimal dose and also its effect on neonatal outcomes, which needs for more research (15).

After this recommendation, several studies were conducted. In 2011, Cetingoz *et al* administered vaginal progesterone (100 mg) to women at risk of preterm delivery including: women with a PTB history, twin gestations and women with uterine anomalies. They observed that although PTB and delivery before 34 wks of gestation decreased significantly for women with a history of PTB compared to placebo group, there was no change in this rate for women with twin pregnancies. The rate was not measurable for women with uterine anomalies due to the low number of the candidates (20). Recently Dodd *et al* in 2013 in Cochrane’s review concluded that although administration of progesterone is associated with benefits in women considered to be at increased risk of preterm birth due to either a prior preterm birth or a short cervix, there is limited information available relating to longer-term infant and childhood outcomes. So they recommended further trials in this field as mentioned by the ACOG before (16). 

In our study, in progesterone group PTB rate was lower than placebo group for women with a previous PTB history, however, in our two subgroups including: 1- women with a previous history of PTB and short cervix and 2- women with uterine anomalies progesterone could not reduce the rates of PTB before 37 wks and 34 wks gestation. (Of course the number of patients with uterine anomalies was relatively low too, however it was higher in our study than in Cetingoz’s). In da Fonseca’s study, there also were very few patients with uterine anomalies and thus they were not studied separately (12). Perhaps there is another mechanism operating which causes PTB in women with uterine anomalies and can not be prevented by progesterone administration. Regarding the neonatal complications, in Cetingoz’s study the length of hospitalization in NICU was significantly shorter in progesterone than placebo group; however there was no difference in neonatal mortality rate (20). 

In this study, we observed no additional benefit with administration of progesterone for prevention of PTB in women who had prior spontaneous PTB and get ultrasound-indicated cerclage for CL <28 mm. It has been shown that a short cervix is a strong predictor of PTB. Definition of short cervix has varied in different studies and cervical length is open to discussion, however, in most studies it is defined as being shorter than 25 mm in 20-24 wks of gestation. It is true that the PTB risk increases as the cervical length decreases. But this risk also varies depending on gestational age which it was measured, number of fetuses, patient symptoms, and prior history of PTB (29). Berghella *et al* in a meta-analysis of 5 controlled randomized clinical trials observed that, cerclage resulted in a reduction in the rate of PTB, neonatal complications and neonatal deaths in women with a previous history of PTB, singleton pregnancy and cervical length shorter than 25 mm (30). 

In a number of studies vaginal or 17-OH progesterone caproate has been used for patients with a short cervix to reduce the PD rate. Defranco *et al* observed that, with progesterone gel (90 mg), there was significant reduction in PTB rate at ≤32 wks of gestation and neonatal complications (admission to NICU and the length of stay in NICU) in patients with a cervical length <28 mm compared with placebo group (19). 

In a multicenter, randomized, double-blind, placebo-controlled trial in 2011, Hassen and colleagues demonstrated that the administration of progesterone gel (90 mg) to women with singleton pregnancy and a short cervix (10-20 mm), starting from 20-23 + 6 wks until 36+6 wks of gestation was associated with a substantial reduction in PTB rate before 35, 33 and 28 wks of gestation, and a significant decrease in RDS rates and the neonatal birth weight <1500 gm (31).

In 2012, Grobman *et al* did not observe any difference in preterm delivery rate in nulliparous women with a cervical length ≤30 mm after the administration of 17-OHP (weekly intramuscular injections of 250 mg) compared with placebo (18). Bergella *et al *showed that the injection of 17-OHP (250 mg IM weekly) starting from 16 to 22+6/7 wks until 36 wks of gestation, had no additional benefit for preventing of PB in women who had prior spontaneous PB and ultrasound-indicated cerclage for CL <25 mm. But in women who did not have cerclage, 17P reduced previable birth and perinatal mortality (32). More recently Alfirevic *et al* in a study on patients with singleton pregnancy, previous spontaneous PTB and a cervical length ≤ 25 mm, observed that vaginal suppository progesterone (200 mg), pessary and cerclage are all equally effective in reducing the PTB rate, perinatal losses and neonatal morbidity (33). 

No randomized controlled trial has been done to compare vaginal progesterone and cervical cerclage directly for PB prevention in women with sonographic short cervix in the mid trimester, singleton, gestation, and previous spontaneous PB. However, recently an indirect meta-analysis by Conde-Agudelo *et al* showed that either vaginal progesterone (90 mg gel, 200 mg capsule or 100 mg suppository, daily) or cerclage are equally effective in PB prevention in these group of women (34). 

Our limitation in this study was the low number of patients especially women with previous spontaneous PB and short cervix, uterine anomalies and uterine myoma. Thus we recommend that another study needs to be conducted with a larger sample size on these subgroups.

## Conclusion

The findings of this study showed that the administration of vaginal suppository progesterone (400 mg, daily) beginning at 16-22 wks of gestation and continued to 36 wks of gestation can significantly reduce the rate of PTB before 37 and 34 wks of gestation among women with previous spontaneous PTB. In addition, the rates of birth weight of ≤2500 gm and ≤1500 gm, RDS and admission to NICU were significantly decreased among infants of women assigned to this treatment compared to placebo. But, we observed no additional benefit of vaginal progesterone for prevention of PTB in women who had prior spontaneous PTB and had received ultrasound-indicated cerclage for CL <28 mm and women with uterine anomalies.

## Note

***Registration ID in IRCT: IRCT201012273386N2 ***

***This article extracted from Residential thesis (Fereshteh Aslebahar)***
